# Reimagining Pediatrics in a World of Artificial Intelligence: Will We Be Empowered or Imperiled?

**DOI:** 10.1016/j.mcpdig.2025.100258

**Published:** 2025-08-28

**Authors:** Shelby Kutty, Yiu-fai Cheung, Sowmya Viswanathan, David A. Danford

**Affiliations:** 1 Academic Programs, BayCare Health System, Clearwater, FL; 2 Department of Pediatrics, the University of Hong Kong and the Hong Kong University-Shenzhen Hospital, China

Fundamental change is coming to pediatrics. The volume of data we could be using to inform clinical care is large and growing to the point that it challenges our capacity to acquire, organize, and apply it all appropriately to our work as pediatricians. Artificial intelligence (AI) is touted as a tool that will enable us to manage this big data to serve our patients’ best interests. Even now, perhaps without our fully recognizing it, AI is increasingly a part of our professional lives. We hope that AI will empower us to improve our care of children, but we harbor concerns over unintended adverse consequences that this technology could bring to pediatric practice.^1^ Therefore, our objective is to make observations from representative recent publications reflecting the current trajectory of medical AI in order to facilitate preparation for new realities in pediatrics. We will consider 5 areas where change fueled by AI is most likely to be impactful: (1) clinical decision support; (2) clinical interactions; (3) diagnosis and imaging; (4) personalized medicine; and (5) ethical concerns. Management of AI to maximize benefit and minimize risk is complicated by entanglements and interdependencies across the various domains in which AI advances take place. We appreciate this cross-domain complexity in many interactions and dependencies, including how AI-mediated developments in diagnosis and imaging will influence the sort of clinical decision support that AI will provide us.

### Clinical Decision Support

Throughout the AI revolution, the primary mission of pediatric practice, excellence in care to the benefit of the patients, will remain intact. To this end, we expect that AI algorithms will enhance clinical decision support (CDS). Currently, CDS identifies best practice, often from clinical trials, systematic reviews, or expert consensus, and a mechanism alerts the clinician through the electronic medical record when it seems best practice is not being followed. Increasingly, the predictive power of AI algorithms will be harnessed to improve CDS. We have already seen isolated examples of how clinicians can be warned when there is a high likelihood of critical illness. For example, one study^2^ looked at 1470 febrile infants, 138 of whom eventually proved to have sepsis, and trained an algorithm requiring only 4 clinical features (urinalysis, white blood cell count, absolute neutrophil count, and procalcitonin) to predict sepsis with very high sensitivity. Had the algorithm been part of CDS to decide when to obtain lumbar puncture, the authors project that 800 fewer lumbar punctures would have been done to find almost all cases of sepsis. The AI powered CDS systems such as this prototype will become more common in pediatric practice, and it is likely that pediatric care will become safer and more efficient as a result.

### Generative AI in Clinical Interactions

Communication is one of the most complicated, and important, aspects of pediatric practice. Until recently, physicians have had major reservations about whether AI was capable of adding value in this critical area. However, major advances in large language models for generative AI are rapidly changing this. The generative pretrained transformer can receive patient input as natural language questions, symptoms, and requests, then encode these, and pass them along to a complex system of neural networks pretrained to optimize the quality of its responses. Decoder neural networks then reverse the encoder processes to deliver natural language output back to the patient. Generative AI shows promise for some challenging tasks like data collection in adolescent mental health. One published example^3^ included 140 adolescents from a study cohort who took part in testing of an interactive app, IDEABot, for collecting longitudinal data on their mood. Acceptance of this app was high and attrition was minimal, even over the 2-3 years of participation. Compliance with elicited prompts was 91%. Such chatbots are potentially scalable for gathering mental health information from patients in the moment, and may enhance our understanding of mood fluctuations. While we do not expect generative pretrained transformer to replace the physician, we will likely see a proliferation of these generative AI systems to support excellence in pediatric practice.

### AI in Imaging and Diagnosis

Images are unstructured data, so AI is well suited to sort through them and provide a clinically useful synthesis, which meets or exceeds a clinician’s ability to process it. One large study of retinopathy of prematurity^4^ included 36,231 fundoscopic images and reported a convolutional neural network discriminated among normal, mildly affected, semi-urgent, and urgent retina, with accuracy comparable to that of expert physicians. The AI has also found application in the recognition of dysmorphic syndromes.^5^ The DeepGestalt algorithm, trained on over 17,000 images representing over 200 syndromes, outperformed expert clinicians for accuracy in generating a differential diagnosis for the test images.

### AI and Personalized Medicine

AI has the potential to revolutionize the way we interpret clinical research. Currently, the model is to use the randomized controlled trial (RCT) to compare the outcomes of different treatment strategies and identify the one which provides, over the entire population, the superior outcome. A single best practice for treating the condition is thereby identified, and recommended for application to all. AI algorithms, however, have the potential to support predictive allocation, in which the best choice for each individual patient is predicted, and care is arranged in accordance with that. In a recent simulation of how this strategy might affect the outcome of surgical management of hypoplastic left heart syndrome^6^, predictive allocation would have resulted in a substantial minority of patients having a better outcome by receiving the care deemed by the RCT to be the inferior overall choice. Clinical trials of predictive allocation will likely confirm the benefits. This approach may prove especially relevant for pediatrics, where performance and interpretation of RCTs is hampered by the special challenges of relatively rare conditions with widely diverse spectra of clinical manifestations.

### Ethical Concerns

Artificial intelligence capable of bringing about these major changes raises ethical concerns^7^, which include how informed consent will be handled for acquisition of the massive amounts of potentially sensitive personal data required to train AI algorithms. There are doubts that most people can be well enough informed about AI to provide meaningful consent. Privacy is especially important in cultures like the United States, where there may be reluctance to simply sign over highly personal information to a process they neither trust nor understand, even when the goals are benevolent. Minorities rightly fear that systemic biases will be incorporated into algorithms that subject them to ongoing disadvantage. Inherent in the ethical use of AI algorithms is achieving and maintaining quality control, but the specific uniform mechanisms that will assure that this is always done are not yet established. Finally, there are legal issues to be resolved in the public interest, including who will own the algorithms, who will be able to profit from them, and who will be made to pay if harm comes to people from their use.

### Conclusion

As AI plays an ever-larger role in pediatrics, we have two general interests to pursue. We must first, in the tradition of Hippocrates, protect our patients from AI-mediated harm, but while accomplishing this we must not withhold from them the benefits that AI brings. Policies will be required to help us manage this very difficult balance of interests. Ideally, we would want a policy that requires meaningful consent be given by parents, assent to be given by children of appropriate age, and protections be in place against misuse of data and violations of privacy. Another fundamental policy would require that quality standards be met when medical AI systems are launched and that updates be required at suitable intervals to assure that quality is maintained over time. We should have a policy that requires evidence that medical AI systems are free from bias against any group, particularly historically vulnerable groups, and that these same groups enjoy equal access to the benefits that AI support is expected to bring to pediatrics. In this way, we might empower pediatrics with the advantages of medical AI systems while minimizing the peril that would come with their unbridled deployment.

## Potential Competing Interests

The authors report no competing interests.
